# The complete chloroplast genome of *Epimedium grandiflorum* C.Morren 1834 (Berberidaceae), a medicinal plant endemic to Japan

**DOI:** 10.1080/23802359.2026.2732724

**Published:** 2026-09-21

**Authors:** Bi-Bo Zhang, Fang-Min Yu

**Affiliations:** ^a^ Beijing Engineering Corporation Limited, PowerChina, Beijing, China; ^b^ State Key Laboratory of Plant Diversity and Specialty Crops, Institute of Botany, Chinese Academy of Sciences, Beijing, China; ^c^ University of Chinese Academy of Sciences, Beijing, China

**Keywords:** *Epimedium grandiflorum*, chloroplast genome, phylogeny

## Abstract

*Epimedium grandiflorum* C.Morren 1834 is a herbaceous perennial medicinal plant native to Japan. Here, we sequenced and assembled its first complete chloroplast genome. The chloroplast genome is 157,151 bp with a total GC content of 38.7%, containing a large single-copy region (89,464 bp), a small single-copy region (17,219 bp), and a pair of inverted repeat regions (25,234 bp). We identified 112 unique genes, including 78 protein-coding genes, 30 transfer RNA genes, and four ribosomal RNA genes. Phylogenetic analysis shows that *E. grandiflorum* formed a clade with *E. trifoliolatobinatum* (Koidz.) Koidz. and *E. sempervirens* Nakai ex F.Maek. The chloroplast genome will benefit future species identification and phylogenetic studies of *Epimedium*.

## Introduction

*Epimedium* L. (Berberidaceae) is a medicinally important genus with about 62 species (Stearn 2002; Ying 2002). This genus is distributed in the Mediterranean region to East Asia and possesses excellent biological activities, such as anti-tumor, osteoporosis, and so on (Ma et al. 2011; Xi et al. 2018). *Epimedium grandiflorum* C.Morren 1834 is one of the most significant medicinal species within *Epimedium* due to its rich content of bioactive constituents (Gani et al. 2023). Notably, *E. grandiflorum* exhibits the highest concentration of icariin (up to 4.4%) among all *Epimedium* species, which contributes to its multiple pharmacological effects, such as anti-inflammatory, antioxidant, and anti-osteoporosis activities (Verma et al. 2022; Gani et al. 2023). This species is a native plant of Japan, characterized by compact rhizome and white, pale yellow, deep rose, red–purple, or violet flowers (Stearn 2002). According to the classification of Stearn (2002), *E. grandiflorum* belongs to section *Macroceras* of subgenus *Epimedium*. However, the complete chloroplast genome of *E. grandiflorum* has not been reported, which restricts further investigations of its phylogenetic position and genomic basis of the discovered pharmacological effects.

In this study, we assembled the chloroplast genome of *E. grandiflorum* for the first time by employing next-generation sequencing technology. Our primary aim was to examine the characteristics of the chloroplast genome and to clarify its systematic position within *Epimedium*. This study not only provides significant chloroplast genome information, but also enhances our comprehension of the evolutionary relationships within *Epimedium*.

## Materials and methods

### Sample sequencing, data assembly, and annotation

**Figure 1.**
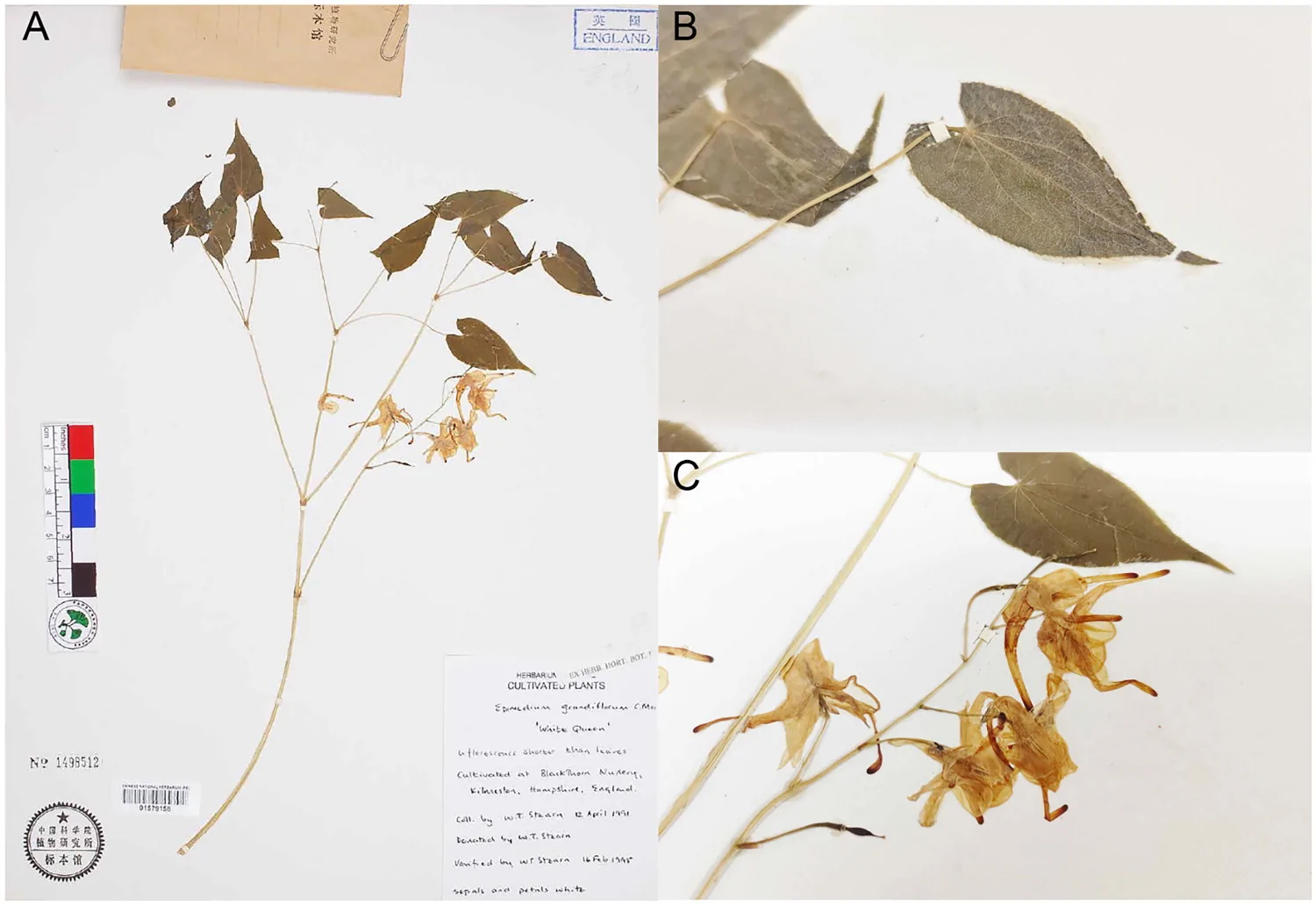
Morphological characteristics of *Epimedium grandiflorum*: (A) plant, (B) leaf, and (C) flower. These specimen photos were taken by Fang-Min Yu at the Herbarium of the Institute of Botany, Chinese Academy of Sciences (PE) Herbarium, China and we have obtained the permission to include the images in this article. *Epimedium grandiflorum* is morphologically characterized by biternately compound leaves with ovate to oblong-ovate leaflets that have finely serrated margins. Its inflorescence is a terminal raceme, with four petals each bearing a slender basal spur, and filaments that are shorter than the petals.

The plant specimen material of *E. grandiflorum* was obtained from Blackthorn Nursery, Kilmeston, Hampshire, UK (51.0312°N, 1.158°W), deposited at the Herbarium of the Institute of Botany, Chinese Academy of Sciences (PE) Herbarium (http://pe.ibcas.ac.cn/ or https://www.cvh.ac.cn/), under voucher number PE 01579158 (contact: Fang-Min Yu, yufangmin@ibcas.ac.cn) (Figure 1). Total genomic DNA was extracted from about 1.5 g of herbarium specimens using a modified cetyltrimethylammonium bromide (CTAB) method (Doyle and Doyle 1987) and purified using the Tiangen Isolation/Extraction/Purification Kit (Tiangen Biotech (Beijing) Co., Ltd., Beijing, China). Short insert of 300–500 bp libraries were prepared for sequencing on the Illumina HiSeq X-Ten platform (San Diego, CA). To obtain high-quality genome sequences, raw reads were processed using Fastp v0.19.7 (Chen et al. 2018) to remove adapter sequences and low-quality reads.

The complete chloroplast genome was *de novo* assembled with SPAdes v3.15.2 (Bankevich et al. 2012). The obtained chloroplast contigs were further visualized and scaffolded to complete circular genomes in Bandage v0.8.1 (Wick et al. 2015), and all genomes were manually adjusted to a consistent orientation using *Epimedium sagittatum* (Siebold & Zucc.) Maxim. 1876 (GenBank Accession No. NC_029428) as the reference. Highly accurate annotation of organelle genomes was performed by using the Organellar Genome GeSeq tool (Tillich et al. 2017), with subsequent manual correction in Geneious v9.0.2 (Kearse et al. 2012). Four chloroplast genomes from *Epimedium sagittatum*, *Epimedium acuminatum* Franch. 1886 (NC_029941), *Epimedium lishihchenii* (NC_029944), and *Epimedium elachyphyllum* Stearn 1997 (NC_053526) were used as reference sequences for their high-quality annotation and structural completeness. The circular chloroplast genome was visualized by using OGDRAW v1.3.1 (Greiner et al. 2019). CPGView was employed to identify both cis-spliced and trans-spliced genes in the genome (Liu et al. 2023). Eventually, the complete chloroplast genome sequence of *E. grandiflorum* was successfully deposited into the NCBI GenBank database (accession number: PX682168).

### Phylogenetic analyses

Except for the newly sequenced chloroplast genome of *E. grandiflorum*, we downloaded chloroplast genome data of 14 closely related species from *Epimedium* from GenBank, which were selected to cover all major phylogenetic clades (Stearn 2002), with *Vancouveria hexandra* C.Morren & Decne. as the outgroup. Multiple sequence alignments of 16 complete chloroplast genome sequences were implemented using MAFFT v7 (Katoh and Toh 2010) with standard parameters, and further adjusted manually in Geneious v8.0.4 (Kearse et al. 2012). Phylogenetic analyses were performed using maximum-likelihood (ML) and Bayesian inference (BI) methods in RAxML v8.2.11 (Stamatakis 2014) and MrBayes 3.2.7 (Ronquist et al. 2012), respectively. RAxML was conducted with the GTR + Γ substitution model, and the fast bootstrap option with 1000 replicates. For BI analyses, two independent runs, each consisting of four Markov Chain Monte Carlo (MCMC) chains, were conducted with one tree sampled for every 1000 generations over 5 million generations. We assessed the convergence of the parameters of the two individual runs in Tracer 1.7.2 (Rambaut et al. 2018). A majority rule (>50%) consensus tree was generated after discarding the first 25% of sampled trees as burn-in.

## Results

**Figure 2.**
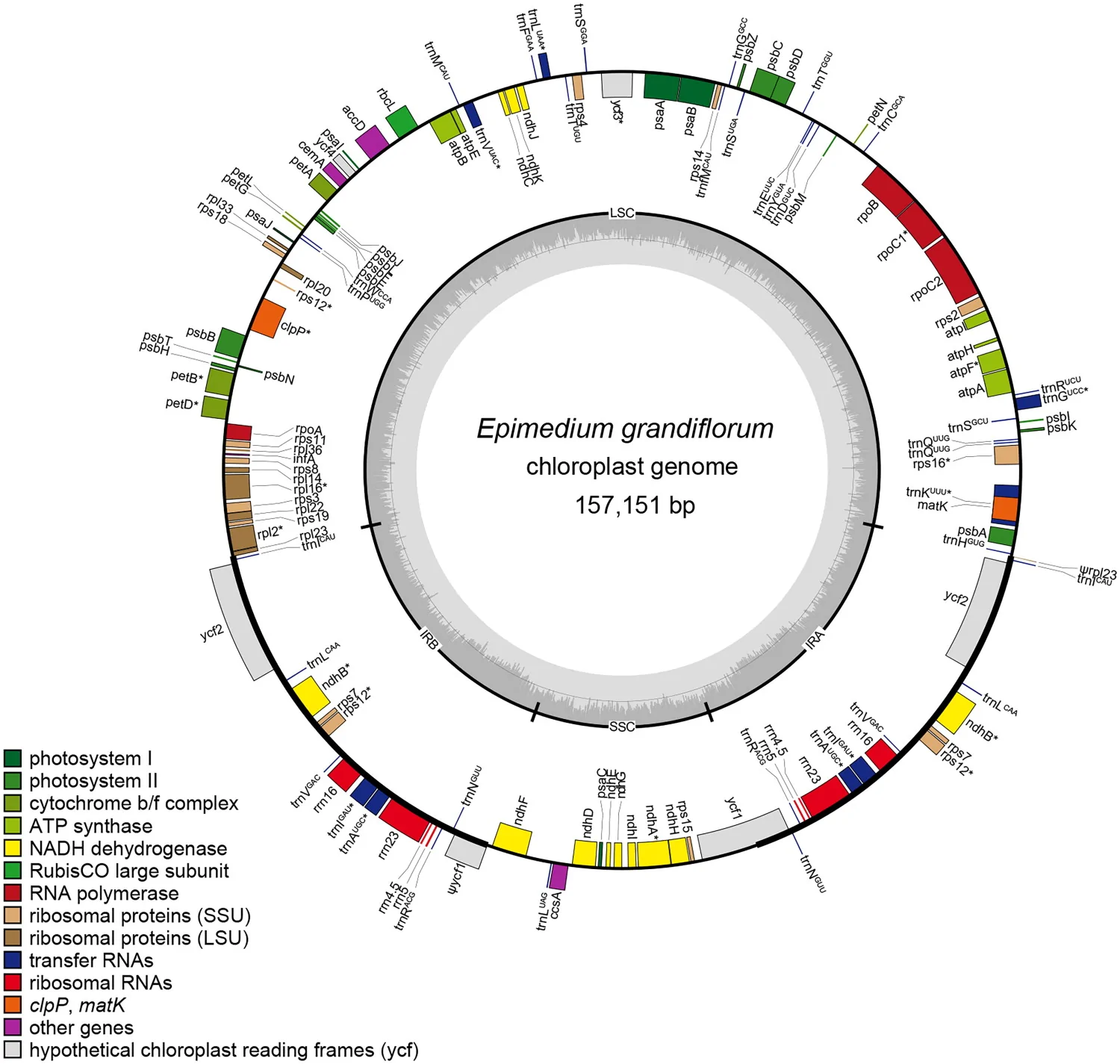
Chloroplast genome map of *Epimedium grandiflorum*. Genes are depicted as differently sized and colored boxes on the outermost circle, with inner and outer boxes representing genes transcribed in clockwise and counter-clockwise directions. The middle circle illustrates changes in GC content at different positions, while the inner circle highlights the regions indicated by the tetrad structure (LSC, SSC, IRa, and IRb) in different colors.

The complete chloroplast genome of *E. grandiflorum* is 157,151 bp in length with an average sequencing depth of 2377.2× (Figure S1). The complete chloroplast genome exhibited typical quadripartite structure (Figure 2), including a large single-copy (LSC) region (89,464 bp), a small single-copy (SSC) region (17,219 bp), and a pair of inverted repeat (IR) regions (25,234 bp each). The overall GC content is 38.7%; the GC contents were 37.3%, 32.8%, and 43.3% in the LSC, SSC, and IR regions, respectively (Table S1). A total of 112 unique genes were identified, including 78 protein-coding genes, 30 transfer RNA (tRNA) genes, and four ribosomal RNA (rRNA) genes, two of which are pseudogenes (*ψycf1* and *ψrpl23*) (Table S2). Two protein-coding genes (*ycf3* and *clpP*) contain two introns, and 16 genes contain a single intron in *E. grandiflorum* (Table S2). Additionally, the chloroplast genome of this species contains 11 cis-splicing genes (*rps16*, *ndhA*, *ndhB*, *rpl2*, *rpl16*, *petB*, *petD*, *atpF*, *clpP*, *rpoC1*, and *ycf3*, Figure S2) and one trans-splicing gene *rps12* (Figure S3).

**Figure 3.**
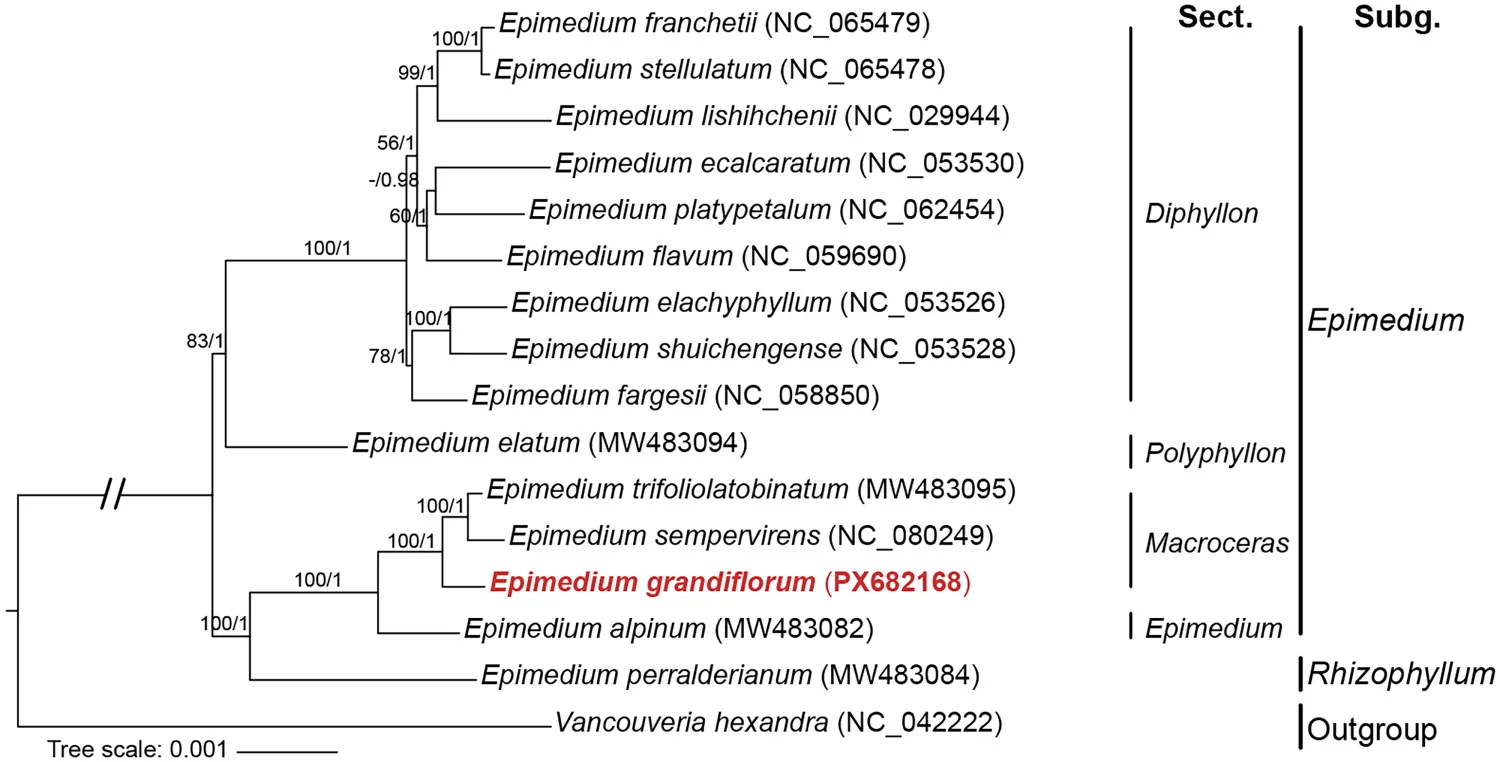
Phylogenetic relationship of *E. grandiflorum* and its 14 closely related species obtained from maximum-likelihood analysis of the complete chloroplast genome dataset. Numbers above branches are bootstrap values (BS ≥ 50%) and posterior probabilities (PP ≥ 0.50), ‘-’ indicates the nodes not supported. The study utilized the following chloroplast genome sequences from the NCBI database, which were publicly available but not yet formally published: *E. franchetii* NC_065479 (direct submission), *E. stellulatum* NC_065478 (direct submission), *E. lishihchenii* NC_029944 (Zhang et al. 2016), *E. ecalcaratum* NC_053530 (unpublished), *E. platypetalum* NC_062454 (unpublished), *E. flavum* NC_059690 (direct submission), *E. elachyphyllum* NC_053526 (Suo et al. 2020), *E. shuichengense* NC_053528 (Yan et al. 2020), *E. fargesii* NC_058850 (direct submission), *E. elatum* MW483094 (unpublished), *E. sempervirens* NC_080249 (unpublished), *E. trifoliolatobinatum* MW483095 (Zhang et al. 2022), *E. alpinum* MW483082 (unpublished), *E. perralderianum* MW483084 (unpublished), and *Vancouveria hexandra* NC_042222 (Sun et al. 2018). The species analyzed in this study (*E. grandiflorum*) is highlighted in bold in the phylogenetic tree.

ML and BI analyses of *Epimedium* generated almost identical topologies based on the complete chloroplast genome sequences (Figure 3). Phylogenetic analysis indicated that *E. grandiflorum* was strongly supported to form a clade with *Epimedium trifoliolatobinatum* (Koidz.) Koidz. 1939 (MW483095) and *Epimedium sempervirens* Nakai ex F.Maek. 1932 (NC_080249) (bootstrap support (BS) = 100%, posterior probability (PP) = 1.0).

## Discussion and conclusions

The complete chloroplast genome of *E. grandiflorum* was firstly sequenced and characterized in the study, uncovering its quintessential quadripartite and circular DNA architecture. The length of this genome is 157,151 bp and its total GC content is 38.7%. A total of 112 unique genes were annotated, including 78 unique protein-coding genes, 30 tRNA genes, and four rRNA genes. This is consistent with the chloroplast genome of other *Epimedium* species (Guo et al. 2022). This foundational understanding of the *E. grandiflorum* chloroplast genome will enhance its applications in comparative genomics and broaden the data available for investigating its pharmacological mechanisms.

The chloroplast phylogenomic analyses show that *E. grandiflorum* in sect. *Macroceras*, congruent with multiple-locus phylogenetic studies (Sun et al. 2005; Zhang et al. 2007, 2014). However, previous studies using limited molecular markers or sparse sampling placed *E. grandiflorum* in conflicting positions within sect. Macroceras with weak nodal support. For example, Sun et al. (2005) using ITS and *atpB*-*rbcL* spacer sequences recovered it as sister to *E. sempervirens*; Zhang et al. (2007) based on *rbcL* gene showed it as sister to *E. trifoliolatobinatum*; and Zhang et al. (2014) using amplified fragment length polymorphism markers supported the sister relationship between it and *E. sempervirens*, while placing *E. trifoliolatobinatum* distantly. In contrast, our phylogenomic analysis of the complete chloroplast genome robustly resolved it as sister to the clade comprising *E. trifoliolatobinatum* and *E. sempervirens* with strong support (Figure 3). Considering the geographic distribution of species within this clade, Japan’s complex topography and climate may have promoted diversification within this lineage. Overall, complete chloroplast genomes enhance phylogenetic resolution in *Epimedium*, and future work with dense taxon sampling will be needed to construct a robust framework.

Overall, this study provides the first complete chloroplast genome for *E. grandiflorum*. This genomic data offer a valuable genomic resource for future research on the species identification, phylogenetic studies, and medicinal applications of *Epimedium*.

## Supplementary Material

Supplemental data.docx

## Data Availability

The genome sequence data that support the findings of this study are openly available in GenBank of NCBI at https://www.ncbi.nlm.nih.gov/ under the accession no. PX682168. The associated BioProject, SRA, and BioSample numbers are PRJNA1497324, SRR39711369, and SAMN61798361, respectively.
