# Supplementary material for: The complete chloroplast genome of Epimedium grandiflorum C.Morren 1834 (Berberidaceae), a medicinal plant endemic to Japan
Source: Mitochondrial DNA B Resour. 2026 Sep 21;11(10):1213–7. doi: 10.1080/23802359.2026.2732724 (PMC13600287; doi:10.1080/23802359.2026.2732724)
Supplement: Supplementary file 1 [file TMDN_A_2732724_SM7897.docx]

**Supplemental data**

**The complete chloroplast genome of *Epimedium grandiflorum* C.Morren 1834 (Berberidaceae), a medicinal plant endemic to Japan**

Bi-Bo Zhang^a^, Fang-Min Yu^b,c*^

^a^Beijing Engineering Corporation Limited, PowerChina, Beijing 100024, China; ^b^State Key Laboratory of Plant Diversity and Specialty Crops, Institute of Botany, Chinese Academy of Sciences, Beijing 100093, China; ^c^University of Chinese Academy of Sciences, Beijing 100049, China

*Correspondence: Fang-Min Yu, yufangmin@ibcas.ac.cn

**Supplementary Figure 1** Coverage depth distribution of the *Epimedium grandiflorum* chloroplast genome. The sequencing depth ranged from 297× to 7991×, with an average depth of 2377.2×.

**Supplementary Figure 2** Schematic map of the cis-splicing genes in *Epimedium grandiflorum* chloroplast genome. Exons and introns are shown in black and white, respectively. The arrow indicates the sense direction of the gene.

**Supplementary Figure 3** Structure of the trans-splicing gene *rps12* in the *Epimedium grandiflorum* chloroplast genome.

**Supplementary Table 1** Assembly and annotation statistics of *Epimedium grandiflorum* chloroplast genome.

| **Features** | **Values** |
| --- | --- |
| Coverage depth (×) | 2377.2 |
| Total size (bp) | 157,151 |
| LSC size (bp) | 89,464 |
| SSC size (bp) | 17,219 |
| IR size (bp) | 25,234 |
| Overall GC content (%) | 38.7 |
| GC content in the LSC (%) | 37.3 |
| GC content in the SSC (%) | 32.8 |
| GC content in the IR (%) | 43.3 |
| Number of unique genes | 122 |
| Number of unique protein-coding genes | 78 |
| Number of unique tRNAs | 30 |
| Number of unique rRNAs | 8 |

**Supplementary Table 2** Summary of characteristics of genes in chloroplast genome of *Epimedium grandiflorum*. The “/” symbol is used to distinguish different copies of the gene.

| **Gene group** | **Gene** | ***Epimedium grandiflorum*** | | |
| --- | --- | --- | --- | --- |
|  |  | **No. copy** | **No. introns** | **No. exon** |
| Subunit Acetyl- CoA-Carboxylate | *accD* | 1 | 0 | 1 |
| ATP synthase | *atpA* | 1 | 0 | 1 |
|  | *atpB* | 1 | 0 | 1 |
|  | *atpE* | 1 | 0 | 1 |
|  | *atpF* | 1 | 1 | 2 |
|  | *atpH* | 1 | 0 | 1 |
|  | *atpI* | 1 | 0 | 1 |
| c-type cytochrome synthesis gene | *ccsA* | 1 | 0 | 1 |
| Envelop membrane protein | *cemA* | 1 | 0 | 1 |
| ATP-dependent protease subunit gene | *clpP* | 1 | 2 | 3 |
| Translational initiation factor | *infA* | 1 | 0 | 1 |
| Maturase | *matK* | 1 | 0 | 1 |
| NADH oxidoreductase | *ndhA* | 1 | 1 | 2 |
|  | *ndhB* | 2 | 1 | 2 |
|  | *ndhC* | 1 | 0 | 1 |
|  | *ndhD* | 1 | 0 | 1 |
|  | *ndhE* | 1 | 0 | 1 |
|  | *ndhF* | 1 | 0 | 1 |
|  | *ndhG* | 1 | 0 | 1 |
|  | *ndhH* | 1 | 0 | 1 |
|  | *ndhI* | 1 | 0 | 1 |
|  | *ndhJ* | 1 | 0 | 1 |
|  | *ndhK* | 1 | 0 | 1 |
| Cytochrome b6/f complex | *petA* | 1 | 0 | 1 |
|  | *petB* | 1 | 1 | 2 |
|  | *petD* | 1 | 1 | 2 |
|  | *petG* | 1 | 0 | 1 |
|  | *petL* | 1 | 0 | 1 |
|  | *petN* | 1 | 0 | 1 |
| Photosystem I | *psaA* | 1 | 0 | 1 |
|  | *psaB* | 1 | 0 | 1 |
|  | *psaC* | 1 | 0 | 1 |
|  | *psaI* | 1 | 0 | 1 |
|  | *psaJ* | 1 | 0 | 1 |
| Photosystem II | *psbA* | 1 | 0 | 1 |
|  | *psbB* | 1 | 0 | 1 |
|  | *psbC* | 1 | 0 | 1 |
|  | *psbD* | 1 | 0 | 1 |
|  | *psbE* | 1 | 0 | 1 |
|  | *psbF* | 1 | 0 | 1 |
|  | *psbH* | 1 | 0 | 1 |
|  | *psbI* | 1 | 0 | 1 |
|  | *psbJ* | 1 | 0 | 1 |
|  | *psbK* | 1 | 0 | 1 |
|  | *psbL* | 1 | 0 | 1 |
|  | *psbM* | 1 | 0 | 1 |
|  | *psbN* | 1 | 0 | 1 |
|  | *psbT* | 1 | 0 | 1 |
|  | *psbZ* | 1 | 0 | 1 |
| Rubisco large subunit | *rbcL* | 1 | 0 | 1 |
| Ribosomal proteins (LSU) | *rpl2* | 1 | 1 | 2 |
|  | *rpl14* | 1 | 0 | 1 |
|  | *rpl16* | 1 | 1 | 2 |
|  | *rpl20* | 1 | 0 | 1 |
|  | *rpl22* | 1 | 0 | 1 |
|  | *rpl23* | 2 | 0/1 | 1/0 |
|  | *rpl33* | 1 | 0 | 1 |
|  | *rpl36* | 1 | 0 | 1 |
| RNA polymerase | *rpoA* | 1 | 0 | 1 |
|  | *rpoB* | 1 | 0 | 1 |
|  | *rpoC1* | 1 | 1 | 2 |
|  | *rpoC2* | 1 | 0 | 1 |
| Ribosomal proteins (SSU) | *rps2* | 1 | 0 | 1 |
|  | *rps3* | 1 | 0 | 1 |
|  | *rps4* | 1 | 0 | 1 |
|  | *rps7* | 2 | 0 | 1 |
|  | *rps8* | 1 | 0 | 1 |
|  | *rps11* | 1 | 0 | 1 |
|  | *rps12* | 2 | 1 | 2/3 |
|  | *rps14* | 1 | 0 | 1 |
|  | *rps15* | 1 | 0 | 1 |
|  | *rps16* | 1 | 1 | 2 |
|  | *rps18* | 1 | 0 | 1 |
|  | *rps19* | 1 | 0 | 1 |
| Conserved Open reading frames | *ycf1* | 2 | 0/1 | 1/0 |
|  | *ycf2* | 2 | 0 | 1 |
|  | *ycf3* | 1 | 2 | 3 |
|  | *ycf4* | 1 | 0 | 1 |
| rRNA genes | *rrn4.5* | 2 | 0 | 1 |
|  | *rrn5* | 2 | 0 | 1 |
|  | *rrn16* | 2 | 0 | 1 |
|  | *rrn23* | 2 | 0 | 1 |
| tRNA genes | *trnA^UGC^* | 2 | 1 | 2 |
|  | *trnC^GCA^* | 1 | 0 | 1 |
|  | *trnD^GUC^* | 1 | 0 | 1 |
|  | *trnE^UUC^* | 1 | 0 | 1 |
|  | *trnF^GAA^* | 1 | 0 | 1 |
|  | *trnfM^CAU^* | 1 | 0 | 1 |
|  | *trnG^GCC^* | 1 | 0 | 1 |
|  | *trnG^UCC^* | 1 | 1 | 2 |
|  | *trnH^GUG^* | 1 | 0 | 1 |
|  | *trnI^CAU^* | 2 | 0 | 1 |
|  | *trnI^GAU^* | 2 | 1 | 2 |
|  | *trnK^UUU^* | 1 | 1 | 2 |
|  | *trnL^CAA^* | 2 | 0 | 1 |
|  | *trnL^UAA^* | 1 | 1 | 2 |
|  | *trnL^UAG^* | 1 | 0 | 1 |
|  | *trnM^CAU^* | 1 | 0 | 1 |
|  | *trnN^GUU^* | 2 | 0 | 1 |
|  | *trnP^UGG^* | 1 | 0 | 1 |
|  | *trnQ^UUG^* | 2 | 0 | 1 |
|  | *trnR^ACG^* | 2 | 0 | 1 |
|  | *trnR^UCU^* | 1 | 0 | 1 |
|  | *trnS^GCU^* | 1 | 0 | 1 |
|  | *trnS^GGA^* | 1 | 0 | 1 |
|  | *trnS^UGA^* | 1 | 0 | 1 |
|  | *trnT^GGU^* | 1 | 0 | 1 |
|  | *trnT^UGU^* | 1 | 0 | 1 |
|  | *trnV^GAC^* | 2 | 0 | 1 |
|  | *trnV^UAC^* | 1 | 1 | 2 |
|  | *trnW^CCA^* | 1 | 0 | 1 |
|  | *trnY^GUA^* | 1 | 0 | 1 |
